# Editorial: Immunopathogenesis and infection characteristics of zoonotic viral diseases

**DOI:** 10.3389/fcimb.2023.1198392

**Published:** 2023-04-14

**Authors:** Muhammad Imran Arshad, Jonas Johansson Wensman, Muhammad Munir

**Affiliations:** ^1^ Institute of Microbiology, Center for Advanced Studies (CAS)/D-8 Research Center, University of Agriculture Faisalabad, Pakistan Academy of Sciences (PAS), Faisalabad, Pakistan; ^2^ Department of Clinical Sciences, Swedish University of Agricultural Sciences, Uppsala, Sweden; ^3^ Department of Disease Control and Epidemiology, National Veterinary Institute, Uppsala, Sweden; ^4^ Division of Biomedical and Life Sciences, Lancaster University, Lancaster, United Kingdom

**Keywords:** zoonosis, one health, host-pathogen, COVID-19, rabies

Emerging and re-emerging zoonotic viral diseases have historically posed a serious threat to animal, environmental, and public health and have impacted economies, trade, and tourism around the world. Highly impactful viral diseases such as COVID-19, SARS, MERS, Ebola, avian influenza and Mpox are associated with multifactorial determinants. Crucially important determinants include pathogen spillover from animals to humans, wide host range, multiple cellular tropisms, and changing host-agent-environment dynamics. These disease-causing factors are cumulatively described as “One Health,” which addresses the intricate interrelationships between human, animal, plant, and environmental health (Arshad et al., 2020; Afzal et al., 2022; Lefrançois et al., 2023). The One Health approach is globally accepted to curtail 60-70% of human infectious diseases associated with animals (zoonosis) as per the WHO-FAO-WOAH-UNEP quadripartite. Therefore, this special issue on “Immunopathogenesis and infection characteristics of zoonotic viral diseases” collates the findings and perspectives on zoonotic viral diseases of public health concern. The aim of this research topic was to recapitulate and present relevant and recent data on zoonotic diseases and to unravel the basic mechanisms of immunopathogenesis. In the post-COVID-19 era, the alarming rapid emergence of pandemics necessitates the investigation of zoonotic aspects (spillover, transmission dynamics, contagiousness, reservoir hosts), virus-host interactions, and the immunogenicity of zoonotic infections. The topic has covered a range of viral diseases such as rabies, COVID-19, poultry coronaviruses, feline panleukopenia virus, and HEV co-infections.

One first study (Khalifa et al.) provided evidence for receptor-mediated spillover of rabies virus in different host species and found the absence of the integrin plexin domain and signaling domain in the integrin β1 (ITGB1) receptor in black fruit bats. It was found that the glycoprotein (G) ectodomain of the rabies virus is indispensable for interaction with ITGB1 in humans, dogs, and bats and for virus entry into different cells/hosts. This molecular docking and structural biology study provided insight into rabies virus transmission dynamics, virulence, and vaccine development insights. Another study (Embregts et al.) demonstrated that rabies virus triggered activation and polarization of human macrophages with upregulation of macrophage-derived antiviral genes (e.g., APOBEC3A, IFIT/OAS/TRIM genes). Furthermore, *in vitro* rabies virus infection stimulated a unique virus-induced polarization of macrophages from M0 to M1, M2a, and M2c phenotypes and antiviral interferon pathways. This study suggests a potential antiviral role for macrophages in controlling the rabies virus at the cellular and molecular levels.

One research (Dai et al.) elucidated the effect of dexamethasone (a steroid) in a combined stress and infectious bronchitis virus (IBV) infection model in chickens. The metabolomics-based study concluded that dexamethasone decreased immunity, caused weight loss, and increased viral load in chicken kidneys in association with increased serum cholesterol, fatty acids, amino acids, and sugar metabolites. This study demonstrated altered persistence and clearance of IBV in the chicken host under induced stress, which has clinical application for the use of dexamethasone during viral infections.

The economic and health losses and mortality associated with COVID-19 are attributed to SARS-CoV-2 virulence, variant emergence, demographics, host characteristics, pre-existing infections, and herd immunity. The increased death rate in COVID-19 patients was significantly correlated with age and co-infections. One of the studies (Sharma et al.) found that mucomycosis co-infection in COVID-19 patients in India was associated with an increased mortality rate and pre-existing co-morbidities such as diabetes, cancer, steroid therapy, and elevated iron levels. At the cellular level, mucosal co-infection in COVID-19 patients was mediated by overexpression of glucose-regulated protein 78 (GRP78), availability of hepcidin-dependent free iron, and compromised host immune responses. SARS-CoV-2 entry into host cells was mediated by the angiotensin-converting enzyme-II (ACE2) receptor, which is primarily expressed in lung epithelial cells, the gastrointestinal tract (GIT), hepatocytes, and the reproductive tract (Ahmad et al., 2021; Khreefa et al., 2023). The SARS-CoV-2 infection and invasion-associated hepatic injuries may predispose individuals to hepatocellular carcinoma (HCC) *via* ischemia/hypoxia, cytokine storm, and antibody-dependent enhancement (ADE) disease mechanisms, as shown by Saeed et al.. This study suggested that the expression of the ACE2 receptor in adipose tissue may lead to the progression of liver injury to non-alcoholic fatty liver disease (NAFLD) and non-alcoholic steatohepatitis (NASH) in COVID-19-affected patients.

Another study (Yi et al.) identified the feline panleukopenia virus (FPV) in giant pandas (*Ailuropoda melanoleuca*) and described its pathogenicity and phylogenetic clusters. The study showed a unique point mutation substitution at G299E residue in the capsid/VP2 protein of FPV, which increased the antigenicity and host range of FPV and posed a threat to the life of the endangered giant panda species. Finally, a case study was reported about a Chinese patient who had co-infections of HEV, *E. coli*, and *Clonorchis sinensis* (Chinese liver fluke, a zoonotic parasite) in biliary drainage. The patient was found to have elevated bilirubin, aminotransferases (ALT, AST), HEV-IgM, HEV-RNA, and a positive bacterial culture.

In conclusion, this Research Topic summarizes findings on rabies virus, FPV, IBV, HEV, SARS-CoV-2, and co-infections in animals and humans, in addition to *in silico* studies that provide evidence for immunopathogenesis, spillover, immune-prophylaxis and clinical insights for the prevention of viral diseases.

## Author contributions

All authors listed have made a substantial, direct, and intellectual contribution to the work and approved it for publication.
